# Psychological predictors of continued ChatGPT use among university students: the roles of AI literacy, trust, and academic self-efficacy

**DOI:** 10.3389/fpsyg.2026.1900190

**Published:** 2026-09-14

**Authors:** Peichen Hu

**Affiliations:** School of Journalism, Fudan University, Shanghai, China

**Keywords:** academic self-efficacy, AI anxiety, AI literacy, continued ChatGPT use, higher education, trust in AI

## Abstract

**Introduction:**

The rapid diffusion of ChatGPT across higher education has outpaced understanding of the psychological factors associated with how far students sustain and intensify their use of it. Drawing on technology acceptance theory, trust-in-automation theory, and Bandura's self-efficacy theory, this study tested a moderated serial mediation model in which AI literacy is associated with continued ChatGPT use through an indirect pathway involving trust in AI and academic self-efficacy, with AI anxiety moderating the literacy-to-trust association.

**Methods:**

Survey data were collected from 450 Chinese university students who had prior experience of using ChatGPT. Students without such experience were ineligible, so the model concerns variation in continued use among existing users rather than the initial decision to take the tool up. Confirmatory factor analysis supported the measurement model, and a full latent-variable structural equation model with Monte Carlo confidence intervals (20,000 draws) tested the hypotheses.

**Results:**

AI literacy was associated with continued use both directly and indirectly through trust and self-efficacy. The hypothesized serial indirect path, from AI literacy to trust to self-efficacy to continued use, was significant. AI anxiety attenuated the literacy-to-trust association, and the index of moderated mediation indicated that the serial indirect effect was weaker at higher levels of anxiety. The pattern was unchanged when continued-use intention and current use intensity were modeled as separate outcomes.

**Discussion:**

The findings position trust and self-efficacy as sequentially ordered correlates rather than parallel ones, and identify anxiety as a boundary condition on that pattern. Because the design is cross-sectional, this ordering is model-specified rather than established temporally; because the sample comprises students who already use ChatGPT, the results speak to the intensity of continued use and not to first-time uptake. Implications for AI literacy education and the design of student-facing generative AI tools are discussed.

## 1 Introduction

Few educational technologies have entered university life as abruptly as ChatGPT. Within months of its release, generative artificial intelligence (AI) moved from novelty to fixture in how students draft essays, debug code, and revise their thinking (Kasneci et al., 2023; Dwivedi et al., 2023). Surveys of student experience report enthusiasm alongside unease about accuracy, dependence, and academic integrity (Chan and Hu, 2023; Chan, 2023; Chan and Zhou, 2023). What remains far less settled is why, among students who have all crossed the threshold into using these tools, some fold them deeply into their studies while others keep them at the margin. Use is not distributed evenly across a campus, and access alone does not explain the gap.

Research on the uptake of ChatGPT has been organized largely by the technology acceptance model (TAM) and the unified theory of acceptance and use of technology (UTAUT), which show that performance expectancy, effort expectancy, and social influence are associated with students' behavioral intention to use the tool (Strzelecki, 2023; Tiwari et al., 2023; Ma et al., 2024; Camilleri, 2024). Because that tradition addresses uptake rather than the intensity of continued use, we draw on it only for the point that bears on the present question: by entering psychological constructs as parallel antecedents of intention, acceptance models describe which beliefs travel with use rather than how those beliefs are ordered relative to one another.

Three psychological constructs recur in this literature as candidate drivers. AI literacy—the knowledge and skills to evaluate, use, and critically appraise AI—is increasingly treated as a foundational competence for the generative AI era (Laupichler et al., 2022; Knoth et al., 2024; Hornberger et al., 2023). Trust in AI shapes whether users are willing to rely on automated output they cannot fully verify (Araujo et al., 2020; Choudhury and Shamszare, 2023; Bedué and Fritzsche, 2021). Academic self-efficacy, students' belief in their capacity to succeed at academic tasks, is a long-standing predictor of how learners engage new tools (Hanham et al., 2021; Altalhi, 2020). Each has been linked to use on its own. What has not been examined is whether they form an ordered psychological sequence rather than a set of independent inputs.

This study advances three propositions that, taken together, address that gap. First, AI literacy operates as a distal correlate whose association with continued use is, in part, statistically accounted for by more proximal states. Second, trust and academic self-efficacy are treated not as interchangeable parallel mediators but as ordered links: literacy is associated with trust in the tool, trust is associated with students' belief that they can use it to learn, and that belief is associated with continued use. We label this the capability-to-cognition-to-behavior pattern and test it as a serial mediation. Third, the literacy-to-trust association is not assumed to be uniform. AI anxiety, apprehension about interacting with AI, may attenuate it, so that even knowledgeable students show weaker trust on which the rest of the chain depends. We therefore model anxiety as a moderator of the first link and test whether it conditions the indirect pattern as a whole. These propositions concern statistical ordering within a hypothesized model, not demonstrated causal sequence.

We test the model with survey data from Chinese university students who already use ChatGPT, a population for whom the tool is both attractive and contested: access requires circumventing regional restrictions, yet use is widespread through virtual private networks and third-party relay platforms. Restricting eligibility in this way narrows the question the study can answer: the outcome is how far students who already use ChatGPT intend to carry on with it and how intensively they use it now, not whether a student becomes a user in the first place. Within that scope the setting offers a demanding test, because sustained use cannot be taken for granted when every session costs deliberate effort. The contribution is threefold. Theoretically, we move acceptance research from a parallel-predictor description toward a model that specifies a hypothesized order among psychological states. Substantively, we provide what is, to our knowledge, the first test of a serial trust-then-efficacy indirect path for generative AI use. Practically, we identify where interventions are most likely to matter: the literacy-to-trust link as the pivot between capability and confidence, and anxiety as a condition on that pivot.

The Chinese higher-education context sharpens these questions. Generative AI has drawn intense policy and institutional attention across the region, and students navigate an environment in which official access to ChatGPT is restricted even as informal use proliferates (Kamalov et al., 2023; Walczak and Cellary, 2023). Continued use in this setting is not the path of least resistance; it requires deliberate effort, which makes the psychological appetite for these tools especially diagnostic. Studying who sustains use, and through what mechanism, therefore offers leverage on the drivers of generative AI engagement that frictionless-access settings may obscure.

## 2 Theoretical background and hypotheses

### 2.1 From adoption to continued use in higher education

In the acceptance tradition, adoption is understood as the formation of behavioral intention and its translation into use (Strzelecki, 2023; Camilleri, 2024). That formulation covers two analytically distinct questions: whether a person becomes a user at all, and how far an existing user carries on. The present study addresses the second. Because eligibility was restricted to students with genuine prior experience of ChatGPT (see Section 3.1), the outcome modeled here is *continued ChatGPT use*, by which we mean the conjunction of continued-use intention and current use intensity among students who are already users. We do not model the initial decision to take up the tool, and we do not treat the present findings as evidence about students who have never used it.

Conceptualizing the outcome as a blend of intention and current behavior follows work that treats post-adoption engagement as a matter of degree rather than a binary state: students may intend to keep using ChatGPT, use it sporadically, or fold it into their routine study practice (Romero-Rodríguez et al., 2023; Bouteraa et al., 2024), and calls to look past intention alone recommend capturing both (Shahzad et al., 2024; Malik et al., 2023). Intention and behavior are nonetheless distinguishable stages in principle, and combining them requires empirical warrant rather than assumption. Theoretically the two remain separate: expectation-confirmation accounts of information-system continuance treat continuance intention as a judgment formed from confirmation and satisfaction, and continued use as a behavioral consequence of that judgment, so that the two can diverge when habit, access constraints, or situational demands intervene (Bhattacherjee, 2001). We retain that distinction conceptually even where these data do not separate the two components empirically, and we report the outcome as a composite only because the measurement model in this sample does not support treating them as distinct factors. Section 4.7 reports the tests we conducted for this purpose: a one-factor specification is compared with a two-factor specification separating intention from current use, and the structural model is re-estimated with the two components as separate outcomes.

Acceptance research has established a robust set of cognitive correlates of generative AI use (Tiwari et al., 2023; Ma et al., 2024; Duong et al., 2023), but typically arrays them side by side as inputs to intention, leaving the associations among them unexamined (Ivanov et al., 2024; Wong et al., 2023). The framework developed below reorganizes three of those constructs into a hypothesized sequence. Hypotheses are numbered by predictor rather than by order of presentation: H1 to H3 concern paths originating in AI literacy (to continued use, trust, and self-efficacy), H4 and H5 paths originating in trust (to self-efficacy and continued use), and H6 the path from self-efficacy to continued use, with mediation hypotheses labeled H7a to H7c and the moderation hypothesis H8.

### 2.2 AI literacy as a distal antecedent

AI literacy refers to the competencies that allow a person to understand, evaluate, use, and critically reflect on AI systems (Laupichler et al., 2022; Knoth et al., 2024). For students, it encompasses knowing what generative models can and cannot do, recognizing their failure modes, and prompting them effectively (Hornberger et al., 2023; Tzirides et al., 2024). Conceptually, literacy maps onto the perceived ease of use and perceived usefulness that anchor TAM: students who understand a tool tend to find it easier to operate and clearer in its benefits (Ma et al., 2024; Lin and Yu, 2023). Empirically, AI literacy and related capability constructs are associated with acceptance and use of AI tools across educational settings (Wang S. et al., 2022; Al-Abdullatif, 2024; AlGerafi et al., 2023). We expect a direct association with continued use, while arguing in subsequent sections that much of literacy's association with continued use is statistically shared with trust and self-efficacy.

**H1**. AI literacy is positively associated with continued ChatGPT use.

### 2.3 Trust in AI as the first mediator

Trust is the willingness to rely on a system under uncertainty and vulnerability, and it is decisive when users cannot fully verify a tool's output (Araujo et al., 2020; Vorm and Combs, 2022). Generative AI sharpens this condition: ChatGPT produces fluent text whose accuracy is not transparent, so reliance hinges on whether students trust it (Choudhury and Shamszare, 2023; Bedué and Fritzsche, 2021). Two associations follow. First, AI literacy should be associated with trust. Understanding how a model works, what it is good for, and where it errs is consistent with calibrated rather than blind reliance, and greater knowledge is linked to lower uncertainty and, in turn, to higher trust (Wong et al., 2023; Nazaretsky et al., 2022). Studies of trust formation report that competence and comprehension covary with higher trust in AI systems (Kim et al., 2021; Montag et al., 2023). Second, trust should be associated with continued use, because reliance is linked to lower perceived risk of acting on AI output (Choudhury and Shamszare, 2023; Mohd Rahim et al., 2022). Trust therefore functions as a proximal mediator between literacy and continued use in the tested model.

**H2**. AI literacy is positively associated with trust in AI.
**H5**. Trust in AI is positively associated with continued ChatGPT use.
**H7a**. Trust in AI mediates the association between AI literacy and continued ChatGPT use.

### 2.4 Academic self-efficacy as the second mediator

Academic self-efficacy is a student's belief in their capacity to organize and execute the actions required to succeed academically (Hanham et al., 2021; Kalender et al., 2020). In Bandura's account (Bandura, 1997), such beliefs draw on mastery experiences, and competently using a capable tool can be one such experience: students who can operate ChatGPT may accumulate small successes that covary with greater confidence in handling academic work (Lu et al., 2022; Gabriel et al., 2020). Literacy should therefore be associated with self-efficacy. Trust should also relate to it. Several considerations motivate placing trust before self-efficacy in the model: belief that ChatGPT can reliably assist learning is part of what is appraised when students judge whether they can use it for academic tasks, so confidence in tool-assisted work has trust in the tool as a referent (Montag et al., 2023; Wong et al., 2023). Evidence that the propensity to trust technology mediates the link between technology self-efficacy and acceptance is consistent with trust operating upstream of efficacy in this domain (Montag et al., 2023). We acknowledge that the reverse ordering is also defensible, since confident learners may more readily extend trust to a new tool; the cross-sectional data tested here cannot adjudicate between these orderings, and we therefore treat the specified sequence as a modeling assumption rather than an established direction. Self-efficacy, in turn, should be associated with continued use, because more confident students tend to integrate new technologies into their study practices more readily (Altalhi, 2020; Hanham et al., 2021; Buabeng-Andoh, 2020).

We measured general academic self-efficacy rather than a ChatGPT-specific efficacy. The claim under test is Bandura's: that competent use of a capable tool feeds a student's broader confidence in academic work, which in turn shapes how they study. A tool-specific mediator would also place ChatGPT in both the mediator and the outcome, making the trust-to-efficacy link close to tautological. The mechanism is worth stating explicitly, since the step from trust in a tool to general academic confidence is not self-evident. Trust in ChatGPT is, on this account, the belief that a resource one is already using will reliably support academic work; a student who holds that belief has one more dependable means of completing assignments, checking understanding, and preparing for assessment. General academic self-efficacy is a judgment about whether one can accomplish such tasks given the resources at hand, and those resources include the tools a student trusts enough to rely on. The path therefore runs through the appraisal of one's working conditions rather than through any direct transfer of confidence from the tool to the self. This reading also bounds the claim: it predicts an association of modest size, which is what we observe, and it does not imply that trust in ChatGPT would raise academic confidence for a student who had no use for the tool. The trade-off remains that the mediator is coarser than a tool-specific scale, and we interpret the second link accordingly: trust in the tool covaries with a general academic confidence that is itself associated with continued use, not with confidence in ChatGPT-supported work specifically.

**H3**. AI literacy is positively associated with academic self-efficacy.
**H4**. Trust in AI is positively associated with academic self-efficacy.
**H6**. Academic self-efficacy is positively associated with continued ChatGPT use.
**H7b**. Academic self-efficacy mediates the association between AI literacy and continued ChatGPT use.

### 2.5 The serial pathway from literacy to continued use

The links specified above imply more than two parallel mediators. If literacy is associated with trust (H2), trust with self-efficacy (H4), and self-efficacy with continued use (H6), then the literacy-use association may be partly accounted for by an ordered indirect path: AI literacy to trust to academic self-efficacy to continued use. This serial specification encodes a hypothesized order rather than a demonstrated one. The model places cognitive appraisal of the tool ahead of self-belief about using it, on the reasoning that trust in the tool is part of what is appraised when students judge their capacity to learn with it (Jia and Tu, 2024; Montag et al., 2023). Treating trust and self-efficacy as interchangeable parallel mediators would not capture this ordering. We therefore test a serial mediation that, to our knowledge, has not previously been examined for generative AI use, while noting that its directional interpretation is a modeling assumption that cross-sectional data cannot confirm.

**H7c**. Trust in AI and academic self-efficacy serially mediate the association between AI literacy and continued ChatGPT use (AI literacy → trust → self-efficacy → continued use).

### 2.6 AI anxiety as a boundary condition

The literacy-to-trust association should not be assumed to hold uniformly. AI anxiety, apprehension, discomfort, or fear in anticipation of interacting with AI, may interfere with the appraisal linking knowledge and trust (Almaiah et al., 2022; Wang Y. et al., 2022). Anxious students may understand a model's capabilities yet remain wary of relying on it, because affective threat can color the interpretation of the same information (Xin and Derakhshan, 2024; Lin and Chen, 2024). The expectation is a negative moderation: the positive association between AI literacy and trust is weaker when AI anxiety is high. Because trust is the first link in the serial path, anxiety should also condition the indirect pattern as a whole, attenuating the indirect association of literacy with continued use through trust and self-efficacy (Budhathoki et al., 2024; Chen, 2022). We test this as a moderated serial mediation.

**H8**. AI anxiety moderates the association between AI literacy and trust in AI, such that the positive association is weaker at higher levels of anxiety; consequently, AI anxiety moderates the serial indirect association of AI literacy with continued use.

Figure 1 presents the full conceptual model.

**Figure 1:**
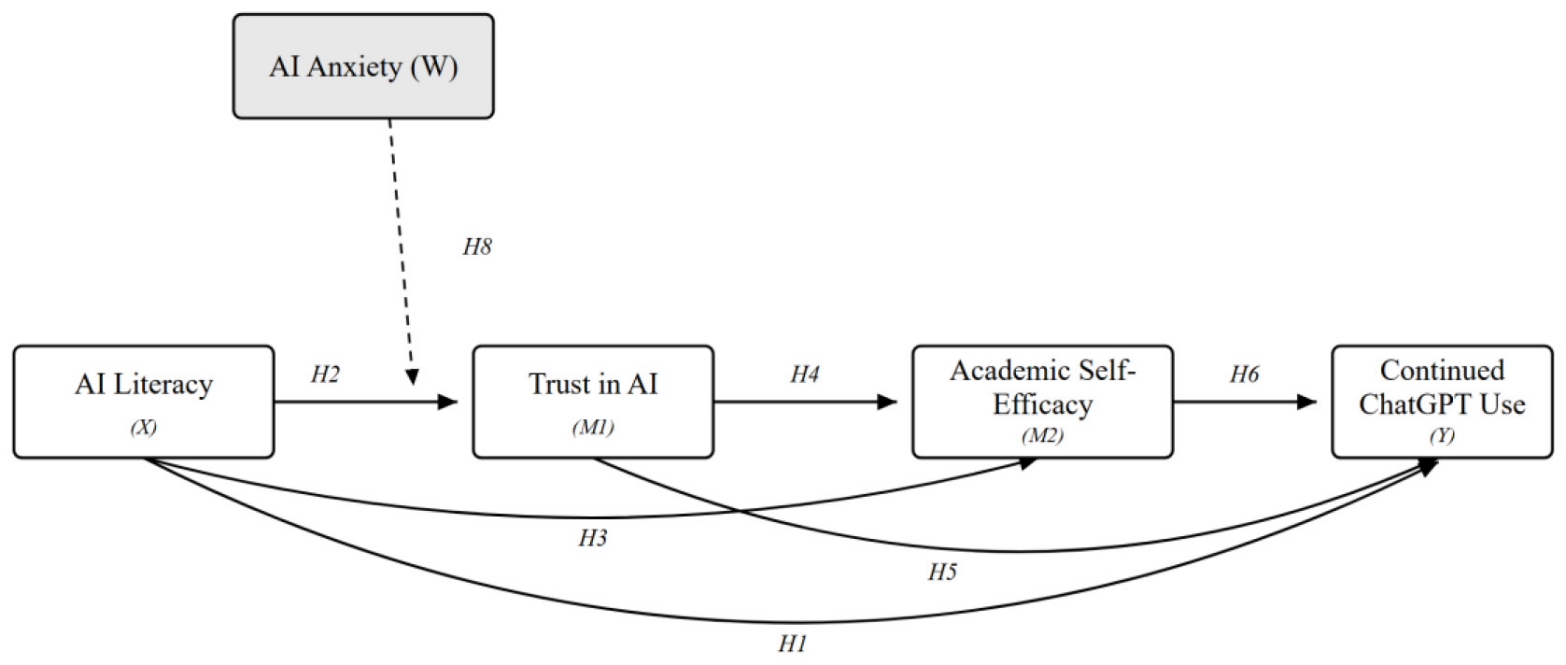
Hypothesized moderated serial mediation model. Solid arrows denote direct paths; the dashed arrow denotes the moderating effect of AI anxiety on the AI literacy–trust path.

Two strands of prior work bear directly on this synthesis. The first treats trust as a correlate of system understanding, reporting that comprehension and perceived competence are associated with greater willingness to rely on AI (Bedué and Fritzsche, 2021; Kim et al., 2021). The second treats self-efficacy as a correlate of technology engagement, reporting that more confident learners take up tools more readily (Hanham et al., 2021; Buabeng-Andoh, 2020). What neither strand has done is join them, asking whether trust is statistically antecedent to the self-efficacy associated with continued use. The serial model tested here makes that join explicit, positioning two well-studied correlates as consecutive links in one indirect path rather than competing explanations.

Positioning anxiety is equally consequential. Prior work has examined apprehension in AI adoption largely as a direct negative predictor of intention or use (Almaiah et al., 2022; Budhathoki et al., 2024). Treating it instead as a moderator of the literacy-to-trust link makes a more precise claim: that affect interferes at the specific juncture where knowledge is appraised into reliance, rather than depressing use uniformly. This specification is testable, and locating the interference at a single stage clarifies where support is most likely to help.

## 3 Materials and methods

### 3.1 Participants and procedure

Participants were 450 students enrolled at universities in mainland China. Because ChatGPT is not officially accessible in the region, eligibility was restricted to students with genuine prior use experience; an initial screening item confirmed that respondents had used ChatGPT through a virtual private network or a third-party relay platform, and those without such experience were excluded before completing the survey.

Prior experience was required for two reasons. First, the trust and use-intensity items presuppose it: trust was measured as willingness to rely on ChatGPT's output, which a non-user would be reporting toward a tool they have not encountered, and the use-intensity items are undefined for them. Second, where access is officially restricted, non-use is largely a matter of whether a student can reach the tool at all rather than of their literacy, trust, or anxiety, so including non-users would confound psychological correlates with access barriers. The outcome is accordingly defined throughout as continued use among existing users, and we do not treat the estimates as generalizing to students who have never used ChatGPT.

Data were gathered through an online questionnaire distributed via university student networks using convenience and snowball sampling. The sample size satisfies common heuristics for structural equation modeling, exceeding both the recommended ratio of cases to estimated parameters and the sample size required for stable estimation of indirect effects in latent-variable structural models. Participation was voluntary and uncompensated.

Of the 450 participants, 56% identified as women and 44% as men. Respondents spanned all undergraduate years and included postgraduate students, and represented science and engineering, social science, and humanities disciplines. Reported prior ChatGPT use ranged from one to twenty months (*M* = 7.96, *SD* = 4.17). Attention-check items were embedded in the questionnaire, and careless responses were removed prior to analysis.

### 3.2 Measures

All constructs were measured with established, multi-item self-report scales adapted to the ChatGPT context. Items originally developed in English were translated into Chinese and back-translated by independent bilingual researchers to ensure semantic equivalence. Unless otherwise noted, items used Likert response formats anchored from strongly disagree to strongly agree.

AI literacy was measured with 12 items on a five-point scale, adapted from instruments developed to assess AI literacy in higher and adult education (Laupichler et al., 2022; Hornberger et al., 2023). Items captured understanding of how generative AI works, awareness of its limitations, and the ability to use and critically evaluate it (e.g., “I can assess the trustworthiness of the information ChatGPT provides”).

Trust in AI was measured with six items on a seven-point scale, adapted from research on trust in automated and AI-based systems (Araujo et al., 2020; Choudhury and Shamszare, 2023). Items assessed willingness to rely on ChatGPT and confidence in its outputs (e.g., “I am willing to rely on ChatGPT's responses when completing academic tasks”).

Academic self-efficacy was measured with eight items on a seven-point scale, adapted from established academic self-efficacy measures used in technology-supported learning research (Hanham et al., 2021). Items assessed confidence in accomplishing academic tasks (e.g., “I am confident I can complete my academic work to a high standard”). As set out in Section 2.4, this is a general rather than a ChatGPT-specific efficacy measure, and the interpretation of the second link in the chain is narrowed accordingly.

AI anxiety was measured with eight items on a five-point scale, adapted from AI anxiety and computer anxiety scales (Wang Y. et al., 2022; Almaiah et al., 2022). Items captured apprehension and discomfort about interacting with AI (e.g., “I feel anxious when I have to rely on AI tools”).

Continued ChatGPT use was measured with seven items on a seven-point scale, adapted from acceptance research on ChatGPT and generative AI (Strzelecki, 2023; Yilmaz and Karaoglan Yilmaz, 2023). Four items assessed intention to keep using ChatGPT for academic purposes and three assessed the frequency and intensity of current use (e.g., “I intend to use ChatGPT regularly in my studies”). Because all respondents were existing users, the intention items refer to continuation rather than to first-time uptake. The empirical justification for combining the two components, and the results of modeling them separately, are reported in Section 4.7.

Gender, year of study, discipline, and months of prior ChatGPT use were included as control variables in the structural analyses. Months of prior use is conceptually distinct from the use-intensity items: it records how long a student has been a user, whereas the items record how heavily they currently use the tool. The two were only weakly related in these data (Section 4.7), and prior use is retained as a covariate throughout.

### 3.3 Analytic strategy

We first estimated a confirmatory factor analysis (CFA) to evaluate the measurement model, assessing reliability (Cronbach's alpha and composite reliability, CR), convergent validity (average variance extracted, AVE), and discriminant validity (the Fornell–Larcker criterion and the heterotrait–monotrait ratio of correlations, HTMT). Model fit was judged against conventional benchmarks: comparative fit index (CFI) and Tucker–Lewis index (TLI) at or above 0.95, root mean square error of approximation (RMSEA) at or below 0.06, and standardized root mean square residual (SRMR) at or below 0.08.

We then tested the structural hypotheses with a full latent-variable structural equation model that represented all five constructs as latent factors indicated by their items, with controls included. The latent interaction underlying the moderation hypothesis (H8) was specified using double-mean-centered product indicators formed by the matched-pair strategy (Lin et al., 2010): the twelve AI literacy items were combined into four three-item parcels and the eight AI anxiety items into four two-item parcels, each indicator was mean-centered, the four matched parcel pairs were multiplied, and the resulting products were mean-centered again. Because each item enters exactly one product, no correlated residuals among product indicators are required. As a sensitivity check the interaction was also estimated with latent moderated structural equations (LMS), which does not rely on product indicators. Indirect effects, including the serial pathway (H7c), were tested with Monte Carlo confidence intervals based on 20,000 draws from the sampling distribution of the relevant parameters; effects were considered significant when the 95% confidence interval excluded zero. To probe the directional assumption built into the serial model, we also estimated an alternative specification that reversed the mediator order (AI literacy → academic self-efficacy → trust → continued use) and compared its fit with that of the hypothesized model. Moderation (H8) was tested with the latent AI literacy × AI anxiety interaction predicting trust, probing it with simple slopes at one standard deviation below and above the mean of anxiety. The moderated serial mediation was evaluated with the index of moderated mediation and its Monte Carlo confidence interval. Because all data were self-reported, we examined common method bias using Harman's single-factor test and an unmeasured latent method construct on which all indicators loaded.

Three further analyses evaluate the outcome specification. First, a one-factor measurement model of the seven outcome items was compared with a two-factor model separating the four continued-use-intention items from the three current-use items, using a chi-square difference test and the latent correlation between the two factors. Second, the full structural model was re-estimated with intention and current use as separate, correlated outcomes, to establish whether the structural paths are stable across the two components. Third, the association between months of prior ChatGPT use and each outcome component was examined to assess the overlap between the covariate and the current-use items. Results are reported in Section 4.7.

## 4 Results

### 4.1 Common method bias

Harman's single-factor test indicated that the first unrotated factor accounted for 31.6% of the variance, below the 50% threshold. We further evaluated common method bias with an unmeasured latent method construct, adding a method factor on which all items loaded alongside their substantive factors. Including this factor improved model fit only trivially (ΔCFI = 0.003), every substantive loading remained significant, and the average variance attributable to the method factor (10.6%) was far smaller than the average substantive variance (48.2%). Common method bias is therefore unlikely to account for the observed relationships.

### 4.2 Measurement model

The confirmatory factor analysis fit the data well, χ^2^(769) = 853.57, χ^2^/df = 1.11, CFI = 0.99, TLI = 0.99, RMSEA = 0.02, SRMR = 0.03. Standardized factor loadings ranged from 0.66 to 0.84 and were all significant. As shown in Table 1, composite reliability ranged from 0.91 to 0.94 and Cronbach's alpha from 0.90 to 0.94, exceeding the 0.70 criterion, and the average variance extracted ranged from 0.56 to 0.61, exceeding 0.50. Reliability and convergent validity were thus supported. Discriminant validity held under the Fornell–Larcker criterion: as Table 2 shows, the square root of each construct's AVE exceeded its correlations with every other construct. The heterotrait–monotrait ratios of correlations were all well below the 0.85 threshold (maximum HTMT = 0.58), providing convergent evidence of discriminant validity.

**Table 1:** Measurement model: reliability and convergent validity.

Construct	Items	λ range	CR	AVE	α
AI literacy	12	0.66–0.83	0.94	0.56	0.94
Trust in AI	6	0.70–0.83	0.91	0.61	0.90
Academic self-efficacy	8	0.70–0.84	0.92	0.60	0.92
AI anxiety	8	0.69–0.80	0.91	0.57	0.91
Continued ChatGPT use	7	0.70–0.83	0.91	0.60	0.91

λ = standardized factor loading; CR = composite reliability; AVE = average variance extracted; α = Cronbach's alpha.

**Table 2:** Means, standard deviations, and correlations among study variables.

Variable	M	SD	1	2	3	4	5
1. AI literacy	3.42	0.67	(0.75)				
2. Trust in AI	4.55	0.89	0.50	(0.78)			
3. Academic self-efficacy	4.78	0.86	0.43	0.53	(0.78)		
4. AI anxiety	2.60	0.76	−0.07	−0.20	−0.02	(0.75)	
5. Continued ChatGPT use	4.90	0.92	0.42	0.50	0.49	−0.06	(0.77)

*N* = 450. AI literacy and AI anxiety used five-point scales; the remaining variables used seven-point scales. Square roots of the average variance extracted are on the diagonal in parentheses. Correlations |r| ≥ 0.10 are significant at *p* < 0.05.

### 4.3 Descriptive statistics and correlations

Table 2 reports means, standard deviations, and intercorrelations. AI literacy, trust, self-efficacy, and continued use were positively and significantly intercorrelated, whereas AI anxiety correlated negatively with trust and was weakly related to the remaining variables. The pattern is consistent with the hypothesized model, in which anxiety acts chiefly on the literacy-to-trust link rather than on the downstream constructs.

### 4.4 Structural model and hypothesis tests

The full latent-variable model fit the data well, χ^2^(1,102) = 1258.41, χ^2^/df = 1.14, CFI = 0.99, TLI = 0.99, RMSEA = 0.02, SRMR = 0.04, and accounted for 40% of the variance in trust, 38% in academic self-efficacy, and 40% in continued ChatGPT use. All six direct paths in this cross-sectional model were significant and in the hypothesized direction (Figure 2; Table 3). AI literacy was associated with continued use directly (β = 0.16, *p* = 0.002), supporting H1. AI literacy was associated with trust (β = 0.50, *p* < 0.001; H2) and academic self-efficacy (β = 0.20, *p* < 0.001; H3). Trust was associated with self-efficacy (β = 0.48, *p* < 0.001; H4) and continued use (β = 0.31, *p* < 0.001; H5). Academic self-efficacy was associated with continued use (β = 0.28, *p* < 0.001; H6). Hypotheses H1 through H6 were thus supported.

**Figure 2:**
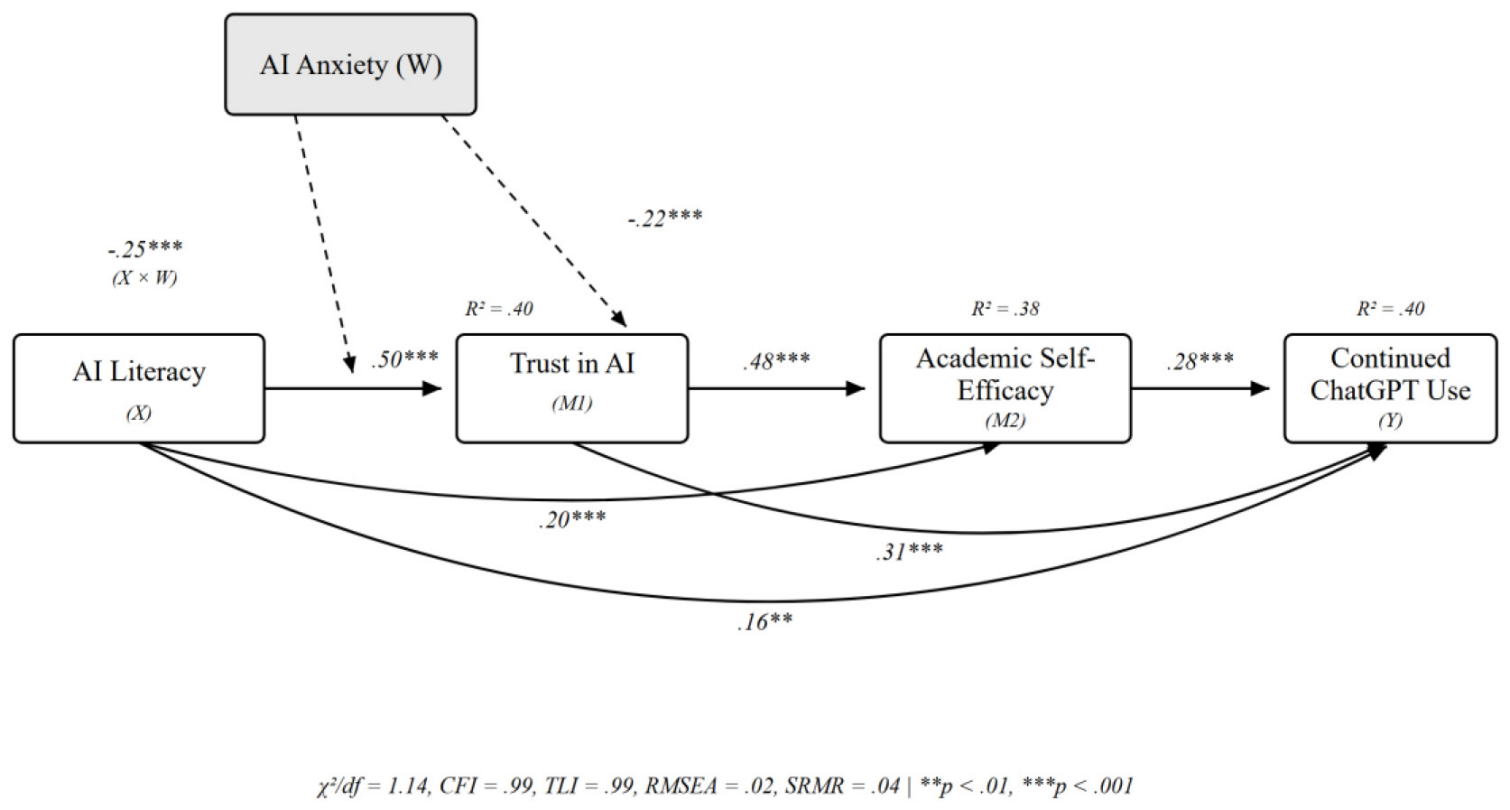
Structural model with standardized path coefficients. Dashed arrows represent the main and interaction effects of AI anxiety. **p < 0.01, ***p < 0.001.

**Table 3:** Standardized direct effects and Monte Carlo indirect effects.

Effect	Estimate	SE	95% CI
Direct effects			
H1 AI literacy → continued use	0.16	(0.05)	[0.06, 0.26]
H2 AI literacy → trust	0.50	(0.05)	[0.40, 0.59]
H3 AI literacy → self-efficacy	0.20	(0.05)	[0.10, 0.30]
H4 trust → self-efficacy	0.48	(0.06)	[0.37, 0.59]
H5 trust → continued use	0.31	(0.06)	[0.19, 0.43]
H6 self-efficacy → continued use	0.28	(0.06)	[0.17, 0.39]
AI anxiety → trust	−0.22	(0.05)	[−0.31, −0.13]
AI literacy × anxiety → trust	−0.25	(0.05)	[−0.35, −0.15]
Indirect effects			
H7a literacy → trust → continued use	0.16		[0.10, 0.22]
H7b literacy → self-efficacy → continued use	0.06		[0.02, 0.09]
H7c literacy → trust → self-efficacy → continued use	0.07		[0.04, 0.10]
Total indirect	0.28		[0.21, 0.36]
Index of moderated mediation (serial)	−0.03		[−0.05, −0.02]

*N* = 450. Estimates are standardized. Indirect effects and the index of moderated mediation were tested with Monte Carlo confidence intervals (20,000 draws); a 95% CI excluding zero indicates significance. Indirect effects were computed from unrounded coefficients and may therefore differ slightly from the products of the rounded direct effects shown, and the separate indirect effects may not sum exactly to the total. All direct effects *p* < 0.001 except the AI literacy → continued use path (*p* = 0.002). Control variables (gender, year, discipline, prior use) were included.

The hypothesized mediator ordering fit better than one alternative. A latent model that reordered the two mediators (AI literacy → academic self-efficacy → trust → continued use) fit the data less well than the hypothesized model on equal degrees of freedom: CFI = 0.984 vs. 0.987, TLI = 0.983 vs. 0.986, and RMSEA = 0.020 vs. 0.018, with a chi-square difference of 36.57. The margin in the incremental indices is small, however: differences of ΔCFI = 0.003 and ΔTLI = 0.003 fall inside the range conventionally treated as practical equivalence, and we therefore do not read this comparison as strong evidence for the hypothesized sequence. What it establishes is the weaker claim that the reverse ordering fits no better. Cross-sectional data cannot establish temporal precedence, and comparing two orderings does not exclude the other specifications that could reproduce these data as well or better; Section 5 returns to this point.

### 4.5 Mediation analysis

Monte Carlo confidence intervals (20,000 draws) supported all mediation hypotheses (Table 3). The indirect association of AI literacy with continued use through trust was significant [indirect effect = 0.16, 95% CI (0.10, 0.22); H7a], as was the indirect association through academic self-efficacy [indirect effect = 0.06, 95% CI (0.02, 0.09); H7b]. The serial indirect path from AI literacy through trust and then self-efficacy to continued use was also significant [indirect effect = 0.07, 95% CI (0.04, 0.10); H7c], consistent with a specification in which trust and self-efficacy function as ordered rather than parallel links, although in cross-sectional data an ordered specification and a parallel one are separated by relative fit rather than by observed sequence. The total indirect effect was substantial [0.28, 95% CI (0.21, 0.36)], and because the direct effect remained significant, trust and self-efficacy jointly accounted for partial, complementary mediation.

### 4.6 Moderated mediation

AI anxiety moderated the AI literacy–trust association in this cross-sectional model (β for the interaction = −0.25, *p* < 0.001), supporting H8. Simple-slope analysis showed that the positive association of literacy with trust weakened as anxiety rose, from 0.76 at one standard deviation below the mean, to 0.50 at the mean, to 0.25 at one standard deviation above (Figure 3). The index of moderated mediation for the serial pathway was negative and significant [−0.03, 95% CI (−0.05, −0.02)], indicating that the indirect association of AI literacy with continued use through trust and self-efficacy was progressively weaker at higher levels of anxiety. The moderated serial mediation was therefore supported. As a supplementary check, because product-indicator approaches to latent interactions can be sensitive to how the products are formed, the interaction was also estimated with latent moderated structural equations (LMS); it remained negative and significant (standardized β = −0.26, *p* < 0.001), and the simple-slope pattern was unchanged.

**Figure 3:**
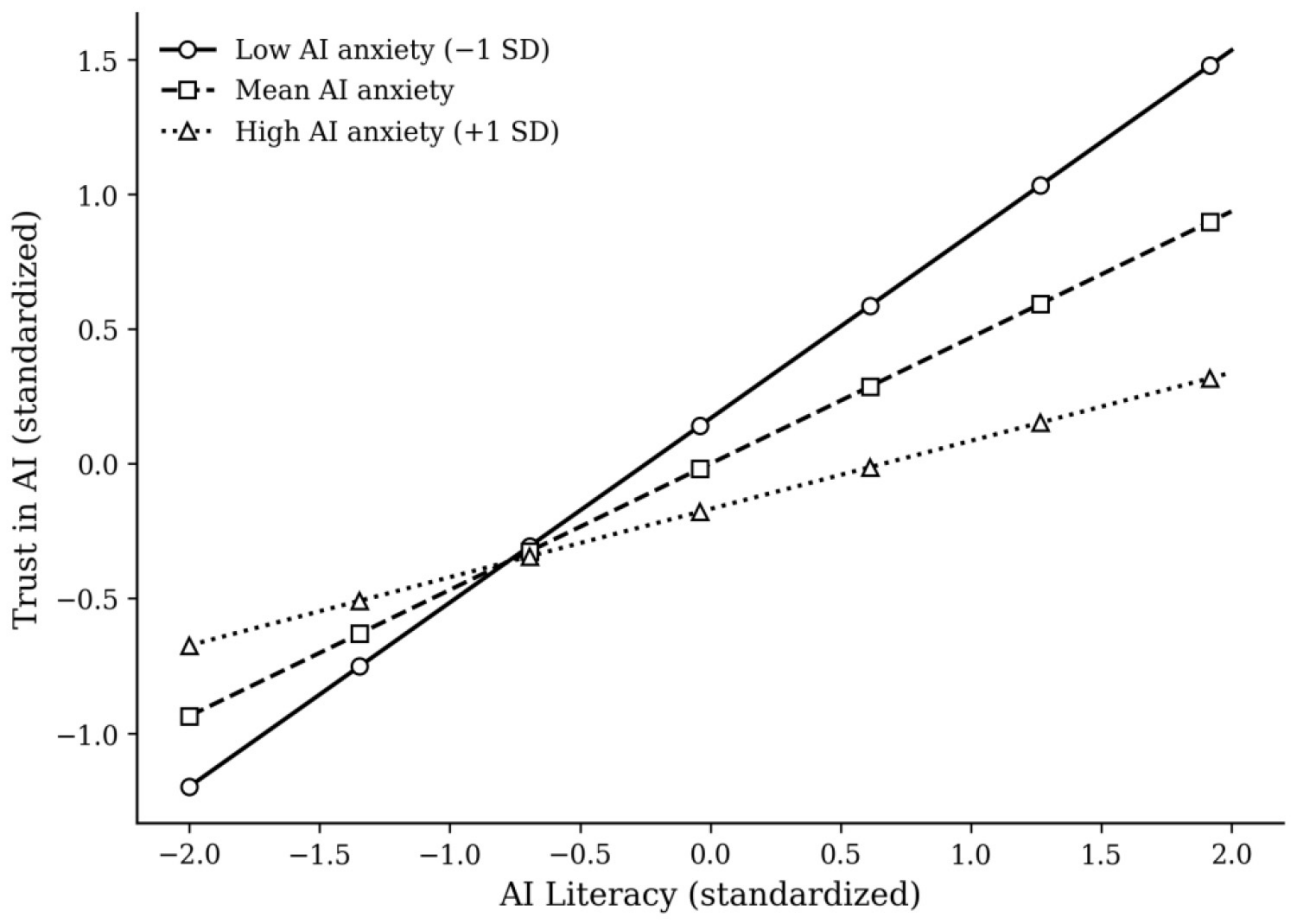
Simple slopes of AI literacy predicting trust in AI at low (−1 SD), mean, and high (+1 SD) levels of AI anxiety.

### 4.7 Robustness of the outcome specification

Because the outcome combines four continued-use-intention items with three current-use items, we tested whether the two components are empirically separable in these data. A two-factor measurement model separating intention from current use did not fit better than a one-factor model, Δχ^2^(1) = 1.51, *p* = 0.219; both fit the data well (one-factor: χ^2^(14) = 10.50, CFI = 1.00, RMSEA = 0.00, SRMR = 0.010; two-factor: χ^2^(13) = 8.99, CFI = 1.00, RMSEA = 0.00, SRMR = 0.009). In the two-factor solution the latent correlation between intention and current use was 0.98 (SE = 0 0.01), which is above conventional discriminant-validity thresholds and indicates that the two components are not distinguishable among these respondents. Among students already using the tool, how much they intend to keep using it and how much they currently use it are close to the same judgment. Treating them as one factor is therefore warranted here, though the warrant is specific to a continuing-user sample rather than a general claim that intention and behavior coincide. The conceptual distinction between the two stages, set out in Section 2.1, is retained irrespective of this empirical result.

We nonetheless re-estimated the full structural model with intention and current use as separate, correlated outcomes. Fit was equivalent to the main model, χ^2^(1,094) = 1247.51, CFI = 0.987, TLI = 0.986, RMSEA = 0.018, SRMR = 0.041, and every structural path retained its sign and significance. AI literacy predicted intention (β = 0.17, *p* = 0.002) and current use (β = 0.14, *p* = 0.009); trust predicted intention (β = 0.32, *p* < 0.001) and current use (β = 0.30, *p* < 0.001); and academic self-efficacy predicted intention (β = 0.24, *p* < 0.001) and current use (β = 0.32, *p* < 0.001). The largest divergence between the two outcomes was 0.08, for the self-efficacy path. The upstream portion of the model was unaffected, since it does not involve the outcome. The substantive conclusions are therefore not an artifact of combining intention with current use.

Finally, months of prior ChatGPT use was only weakly related to either outcome component, *r* = −0.10 with the intention items and *r* = −0.13 with the current-use items. The covariate and the current-use items are thus not redundant: duration of use carries different information from current intensity of use, and the negative sign indicates, if anything, that longer-standing users reported slightly lower current intensity. Months of prior use is retained as a control in all models reported above.

## 5 Discussion

This study asked how AI literacy relates to continued ChatGPT use among students who already use the tool, and whether trust and academic self-efficacy function as an ordered psychological sequence rather than as a set of parallel correlates. The results were consistent with the specified sequential model. AI literacy was associated with continued use both directly and indirectly through trust and self-efficacy, and the serial indirect path from literacy to trust to self-efficacy to continued use was significant. AI anxiety conditioned the first link, attenuating the literacy-to-trust association and, with it, the downstream indirect path. Throughout, we describe associations within a cross-sectional model and do not claim a demonstrated causal sequence.

The primary contribution is to reframe how acceptance research treats psychological correlates of generative AI use. Models in the TAM and UTAUT traditions have established that trust, capability, and confidence each travel with use, but they typically enter these constructs as parallel antecedents of intention (Strzelecki, 2023; Tiwari et al., 2023; Ma et al., 2024). Our findings in this sample are consistent with the hypothesized ordering of two of them. Trust and self-efficacy occupied distinct positions in the modeled sequence: literacy was associated with trust, trust with self-efficacy, and self-efficacy with continued use. Specifying this order moves acceptance research from describing the correlates of use toward modeling how they are arranged, answering recent calls to examine the associations linking acceptance variables (Ivanov et al., 2024; Wong et al., 2023).

The serial indirect path (H7c) is the study's sharpest result. It is consistent with a capability-to-cognition-to-behavior account in which an objective competence is statistically linked to action through intermediate states: understanding of the tool, trust in it, and belief that one can succeed academically are arranged as ordered correlates of continued use. This ordering connects three literatures that have largely run in parallel. It links AI literacy research (Laupichler et al., 2022; Knoth et al., 2024), which has concentrated on conceptualization and measurement, to a behavioral outcome; it positions trust as the pivot through which competence is associated with motivational states (Araujo et al., 2020; Choudhury and Shamszare, 2023); and it extends Bandura's view of self-efficacy by suggesting that, for tool-mediated tasks, confidence in oneself is statistically downstream of trust in the tool within this model (Hanham et al., 2021; Montag et al., 2023). That trust is associated with self-efficacy (H4) is a noteworthy link in its own right: in the tested model, general confidence in one's academic capacity covaries with belief that ChatGPT can be relied upon. Because the efficacy measure is general rather than ChatGPT-specific, this link should be read as connecting trust in the tool to a broad academic confidence, not as showing that trust raises confidence in ChatGPT-supported work specifically; a tool-specific efficacy measure would be needed to test the narrower claim, and the directionality of these links is in any case assumed by the model rather than established by the design. The mechanism set out in Section 2.4, on which trust in a relied-upon tool enters a student's appraisal of the resources available for academic work, is the reading we intend, and it is consistent with the modest size of the coefficient.

The moderation findings refine the account by identifying a boundary condition. Consistent with H8, AI anxiety was associated with a weaker literacy-to-trust link, and the negative index of moderated mediation showed that the serial indirect path was attenuated at higher levels of anxiety. This result qualifies an implicit assumption in much literacy-focused work, that building competence is sufficient (Wang S. et al., 2022; Tzirides et al., 2024). Among more anxious students, literacy was associated with trust less strongly, so the indirect path toward continued use was attenuated at its first link. The pattern aligns with evidence that affective apprehension relates to engagement with AI independently of, and sometimes against, what learners know (Almaiah et al., 2022; Wang Y. et al., 2022; Xin and Derakhshan, 2024). It suggests that affect and cognition interact at a specific juncture, the appraisal linking understanding and reliance, rather than acting as one more parallel correlate of intention.

Two further features merit comment. First, that AI anxiety related strongly to trust but only weakly to self-efficacy and continued use directly suggests its association operates through a particular gateway rather than diffusely; it is best captured as a moderator of the literacy-to-trust link rather than as a global correlate. Second, the persistence of a significant direct path from literacy to continued use indicates that literacy is also associated with continued use through routes not captured here, plausibly perceived usefulness or effort expectancy (Ma et al., 2024; Camilleri, 2024), a reminder that the serial path complements rather than replaces established acceptance mechanisms.

It is useful to situate these results against the parallel-mediator models that dominate the literature. Studies entering trust and confidence as simultaneous predictors can reproduce the bivariate associations observed here, yet they cannot distinguish a model in which one state is statistically antecedent to another from one in which both arise independently (Tiwari et al., 2023; Polyportis and Pahos, 2024). The significant serial path, together with the partial direct effect, indicates that the present data are at least as well described by an ordered specification as by a simultaneous one. This does not overturn parallel-predictor findings so much as offer an alternative reading: what appears to be two independent correlates may be one indirect path observed at two points. Distinguishing the two readings conclusively requires longitudinal data.

The results also speak to debates about what AI literacy is for. If literacy mattered only as a proxy for ease of use, its association with continued use should be largely direct. Instead, a substantial share of that association was shared with trust and self-efficacy, implying that literacy is relevant in part because it relates to how students appraise and engage the tool, not merely how easily they operate it. This positions AI literacy as a psychological as well as a technical competence, consistent with conceptualizations that fold critical evaluation and disposition into the construct (Laupichler et al., 2022; Knoth et al., 2024).

The findings also carry implications for how universities cultivate productive AI use, offered with the caveat that they rest on cross-sectional associations among students who are already users. Because trust occupies the pivot of the modeled chain, AI literacy instruction might aim not only at skills but at calibrated trust. Teaching students what generative models do well, where they fail, and how to verify their output may matter more than coverage of features alone, because such calibration is what is most closely associated with the warranted reliance linked to downstream confidence (Hornberger et al., 2023; Wong et al., 2023). Literacy programs that stop at operation, without addressing trust, may leave the chain weak at its most load-bearing link.

The moderation result points to a second, often neglected target: anxiety. Interventions that raise competence while ignoring affect may underperform among precisely the students who most need support, since higher anxiety is associated with a weaker literacy-to-trust link (Almaiah et al., 2022; Lin and Chen, 2024). Low-stakes, scaffolded encounters with ChatGPT, such as supervised practice, worked examples, and explicit normalization of uncertainty, may reduce apprehension enough for knowledge to be expressed as trust. For designers of student-facing generative AI tools, the implication is that transparency features that make a system's reasoning and limitations legible (Vorm and Combs, 2022) may help both by supporting calibrated trust and by easing the anxiety associated with its absence. Finally, because self-efficacy is the proximal correlate of continued use in the model, framing AI tools around students' academic goals, and surfacing small early successes, may consolidate the confidence associated with sustained use (Hanham et al., 2021; Altalhi, 2020). These implications concern how existing users are supported; the study provides no basis for recommendations about encouraging first-time uptake.

One implication of the moderation result deserves separate comment, because it can be read more pessimistically than the data warrant. AI anxiety was essentially unrelated to continued use directly (*r* = −0.06, ns), so the moderation does not say that anxious students use ChatGPT less. It says something narrower: among students higher in anxiety, knowing more about AI is associated with trusting it less strongly than the same knowledge is among students lower in anxiety, and the simple slopes make the gradient visible (0.76 at −1 SD, 0.50 at the mean, 0.25 at +1 SD). The slope at high anxiety is attenuated rather than absent, so the pattern is one of diminished return on literacy, not of literacy losing all purchase. Two readings remain open and this design cannot separate them. On the first, anxiety interferes with the appraisal by which knowledge becomes reliance, in which case literacy instruction alone would be a poor instrument for anxious students and the affective barrier would need addressing first or alongside it. On the second, students high in anxiety may already hold accurate knowledge whose implication they decline to act on, in which case a shallower literacy-to-trust slope reflects calibration rather than obstruction, and treating it as a deficit to be corrected would be presumptuous. We flag rather than resolve this. The practical inference from a weaker slope is not that anxious students cannot be reached, but that reaching them through literacy alone should not be assumed to work.

Several limitations qualify these conclusions and point toward future work. First, the design was cross-sectional. All five constructs were measured on a single occasion, so temporal precedence is imposed by the model rather than observed, and unmeasured common causes cannot be ruled out. The better-fitting hypothesized ordering in Section 4.4 is informative but not decisive, and the point deserves more than a clause. That comparison rules out one rival specification, not the class of them. Cross-sectional covariance data are typically consistent with many structurally different models: specifications with reciprocal paths between trust and self-efficacy, specifications in which an unmeasured common cause such as general technology optimism or conscientiousness generates the associations among all three constructs, and specifications that move the outcome to a different position in the chain. Some of these are statistically equivalent to the model reported here and cannot be distinguished by fit at all. The margin involved is in any case small: ΔCFI = 0.003 and ΔTLI = 0.003 fall inside the range ordinarily treated as practical equivalence, so the comparison establishes only that the reverse ordering fits no better, which is a weaker claim than it may appear. Statements such as “literacy is associated with trust, which is in turn associated with self-efficacy” describe an ordered statistical pattern, not a claim that changing one construct would change the next. Longitudinal designs with at least three waves, and experiments manipulating literacy or trust directly, are needed to license causal claims about the pathway.

Second, eligibility was restricted to students with prior ChatGPT experience. The sample therefore cannot speak to the initial decision to take up the tool, and the findings should not be read as a model of adoption in that sense. Where access requires a virtual private network or a relay platform, students who qualify are also likely to differ from those who do not, being plausibly more technically resourceful, more willing to tolerate the friction and uncertainty involved in working around access restrictions, more inclined to seek out unfamiliar study tools, and more favorably disposed toward the tool before they ever used it. This is a strongly self-selected group rather than a cross-section of Chinese undergraduates, and the selection operates on characteristics that are themselves plausible correlates of AI literacy, trust, and anxiety. Such selection restricts the range of the constructs at issue and would generally attenuate the associations observed, though it could also inflate them along any path on which the selecting characteristic acts. Extending the model to first-time uptake would require sampling non-users and measuring their intentions toward a tool they have not encountered, which we regard as a distinct study.

Third, the outcome combined continued-use intention with self-reported current use. Section 4.7 shows that the two are not empirically separable in this sample and that the structural paths are stable when modeled separately, but their near-unity correlation is itself a consequence of sampling existing users and does not imply that intention and behavior coincide in general. Both components are self-reported, and self-reported frequency is an imperfect proxy for actual use; behavioral traces such as platform logs would provide a sterner test of the final link.

Fourth, the model deliberately centered three constructs and one moderator; other variables, such as perceived usefulness, social influence, perceived risk, or institutional policy (Chan, 2023; Moorhouse et al., 2023), are also associated with use and could be integrated into the model. Fifth, the academic self-efficacy measure is general rather than ChatGPT-specific, which as discussed above narrows what the trust-to-efficacy link can be taken to show; a study including both general and tool-specific efficacy measures would be able to separate these interpretations. Finally, common method bias was addressed with both Harman's single-factor test and an unmeasured latent method construct, neither of which indicated a serious problem. These are statistical diagnostics, however, and they cannot remove the design features that generate the concern. Every construct was measured by self-report, in one questionnaire, on one occasion, so shared response tendencies, transient mood, and consistency motives remain plausible contributors to the observed covariances; a method factor accounting for 10.6% of indicator variance bounds that possibility rather than eliminating it, and the bound itself rests on the assumption that method variance is adequately captured by a single latent factor. Procedural separation of measurement, a purpose-collected marker variable, and non-self-report indicators of use such as platform logs would each address the problem at its source rather than after the fact.

Future research might also ask whether the serial chain operates differently across tasks and disciplines, whether the trust-to-efficacy link strengthens with experience, and whether anxiety's moderating role diminishes as generative AI becomes routine. Each question follows naturally from the process view advanced here.

## 6 Conclusion

Understanding why some university students carry ChatGPT deep into their studies while others keep it at the margin requires more than cataloging its correlates. Among students who already use the tool, this study found that AI literacy is associated with continued use in a pattern consistent with an ordered psychological sequence: it relates to trust in the tool, trust to academic self-efficacy, and self-efficacy to sustained use. In this model, trust and self-efficacy are treated not as interchangeable parallel correlates but as successive links, and AI anxiety conditions the sequence at its origin by weakening the literacy-to-trust association. By specifying this moderated serial pattern, the study reframes psychological correlates of generative AI use as an ordered set rather than a list, and it points to where educational effort may matter most: cultivating calibrated trust and managing anxiety so that what students know about AI can be reflected in how they use it. Because the evidence is cross-sectional and drawn from existing users, the ordering is a modeling assumption that longitudinal work should test, and the account applies to sustained engagement rather than to first-time uptake. As generative AI settles into the fabric of higher education, accounts of use that examine ordered associations, not prediction alone, may be better guides to supporting students well.

## Data Availability

The raw data supporting the conclusions of this article will be made available by the authors, without undue reservation.
